# Relationship between pulmonary function and physical performance among community‐living people: results from Look‐up 7+ study

**DOI:** 10.1002/jcsm.12485

**Published:** 2019-12-04

**Authors:** Francesco Landi, Sara Salini, Maria Beatrice Zazzara, Anna Maria Martone, Sofia Fabrizi, Mariangela Bianchi, Matteo Tosato, Anna Picca, Riccardo Calvani, Emanuele Marzetti

**Affiliations:** ^1^ Department of Geriatrics, Neurosciences and Orthopedics Catholic University of the Sacred Heart Rome Italy

**Keywords:** Pulmonary function, Physical performance, Sarcopenia

## Abstract

**Background:**

While respiratory muscle strength is recognized to decline with aging process, the relationship between sarcopenia and pulmonary function remains to be studied. The present study was undertaken to provide a better insight into the comprehension of the relationship between pulmonary function and muscle function (strength and physical performance) using an unselected sample of subjects assessed during the Longevity Check‐up 7+ project.

**Methods:**

Look‐up 7+ is an ongoing cross‐sectional survey started in June 2015 and conducted in unconventional settings (i.e. exhibitions, malls, and health promotion campaigns) across Italy. Candidate participants are eligible for enrolment if they are at least 18 years of age and provide written informed consent. Muscle strength was assessed by handgrip strength test, and physical performance was evaluated by chair stand test. Spirometer analysis was performed using the AirSmart system, and the largest forced vital capacity (FVC), forced expiratory volume in 1 s (FEV1), and peak expiratory flow (PEF) values were collected.

**Results:**

The mean age of 925 subjects participating in the Longevity check‐7+ surveys and receiving the spirometer evaluation was 55.6 years (range from 18 to 98 years), and 501 (54%) were women. Overall, both in male and female participants, FVC, FEV1 and PEF positively correlated with handgrip strength and chair stand tests. The receiver operator characteristic curve analysis revealed that the areas under the curves for FVC, FEV1, and PEF were 0.79, 0.80 and 0.80, respectively.

**Conclusions:**

The results clearly show that pulmonary function was positively associated with handgrip strength and chair stand tests. Based on this observation, muscle strength, physical performance, and pulmonary function should be recommended as the method of choice for the early detection of individuals at risk of probable sarcopenia and at the same time to better characterized the severity of sarcopenia status.

## Introduction

Advancing age is linked with important changes in body composition, the most remarkable of which, sarcopenia, is a major cause of physical function decline, disability, and mortality.^1^, ^2^ Some studies have assessed the changes in muscle function during the aging process documenting a substantial age‐related decline in both muscle mass and physical performance.^3^, ^4^, ^5^ In general, muscle strength (as measured by handgrip test) and physical performance (as measured by chair stand test) were both stable in the first decades of adulthood and start to decrement in the middle years (45+) and late adulthood. In particular, individuals with more than 75 years lose >30% in muscle strength and physical function.^6^

Similarly, maximal inspiratory and expiratory muscle strength is recognized to decline with advancing age,^7^, ^8^ and this age‐related reduction in respiratory function is correlated to sarcopenia of the respiratory muscle.^9^ In particular, pulmonary functions, such as the forced vital capacity (FVC), the forced expiratory volume in 1 s (FEV1), and the peak expiratory flow rate (PEF), decrease with aging due to modifications in elastic recoil and thorax compliance.^10^, ^11^

Previous studies described that reduced muscle function was correlated with decreased pulmonary function in subjects with lung disease, such as chronic obstructive pulmonary disease.^12^, ^13^, ^14^ Recently, Bahat *et al*.^15^ demonstrated that muscle strength, assessed by handgrip strength, was positively associated with maximal inspiratory and expiratory muscle strength. However, there is little evidence about how muscle strength and physical performance are connected to pulmonary function in asymptomatic subjects without a clear diagnosis of pulmonary disease.

The present study was, therefore, undertaken to provide a better insight into the comprehension of the relationship between pulmonary function and muscle function (strength and physical performance) using an unselected sample of subjects assessed during the Longevity Check‐up 7+ project.

## Materials and methods

We used data from the Longevity Check‐up 7+ (Look‐up 7+) study, an initiative developed by the Geriatric Medicine Department of Catholic University of Rome and specifically designed with the aim of encouraging a healthy lifestyle in the general population. Subjects visiting public environments (i.e. exhibition and shopping centre) or subjects adhering to prevention campaigns have been screened using a specific questionnaire on lifestyle and performed a brief check‐up.

The Look‐up 7+ study protocol has been described in detail elsewhere.^6^, ^16^ Participants were designated as eligible for the check‐up if they were at least 18 years of age and provided written informed consent. Exclusion criteria were self‐reported pregnancy, an inability to complete the physical performance test, refusal of the blood capillary check, or an inability to give written informed consent. Within the context of the National Campaigns for cardiovascular prevention,^16^ the Catholic University of Sacred Heart Ethical Committee ratified the study protocol.

### Study Sample

Between 1 June 2015 and 30 November 2018, we enrolled 10 644 individuals in different surveys and Italian cities adhering the national campaign, named “Longevity Check‐up 7+” (Look‐up 7+) and promoted by the Catholic University of Rome. For the present study, 925 subjects who performed the spirometer assessment were included.

### Data collection

All people who accepted to be screened underwent individual assessments consisting of a brief questionnaire, the measurement of objective cardiovascular health metrics, and the evaluation of anthropometric parameters (height and weight) and functional performance (lower extremity muscle power). In particular, the Look‐up 7+ visit is structured to collect the following information and data: informed consent, lifestyle interview (smoking, eating habits, physical activity, and previous screening performed), blood pressure measurement, weight and height (body mass index calculation) assessment, and cholesterol and glucose measurements.^16^, ^17^

Smoking habit was categorized as current or never/former smoker. Body weight was measured through an analogue medical scale. Body height was measured using a standard stadiometer. BMI was defined as weight (kg) divided by the square of height (m). Healthy diet was considered as the consumption of at least three portions of fruit and/or vegetables per day.^18^ Regular participation in physical activity was considered as involvement in exercise training at least twice a week. Cholesterol was measured from capillary blood samples using changing reagent strips based on a reflectometric system with the portable device MultiCare‐In.^19^ Random blood glucose was measured from capillary blood samples using changing reagent strips based on an amperometric system with the portable device MultiCare‐In.^19^ Blood pressure was measured with an electronic sphygmomanometer according to recommendations from international guidelines.^20^

### Muscle strength and physical performance assessment

Muscle strength was assessed by handgrip strength test, which was measured by using a dynamometer (North Coast Hydraulic Hand Dynamometer; North Coast Medical, Inc, Morgan Hill, CA). Participants performed one familiarization trial and one measurement trial with each hand, and the result from the stronger side was used for the analyses.

During the Look‐up 7+ screening visit, participants' physical performances were evaluated by chair stand test. Subjects were asked to stand up from a chair with their arms folded across the chest five times in a row as quickly as possible. A standard armless chair is used, usually 43–47 cm in height. The back of the chair is stabilized against a wall to ensure safety and stability. The time taken to perform the task is measured using a handheld stopwatch; increased time reflects poorer performance. The five‐repetition chair stand test is simple to use, reliable, and valid measure of physical function in adult and older people, including those with musculoskeletal or neurological conditions. Poor performance on this test highlights mobility problems and is associated with subsequent disability.^21^

### Pulmonary function assessment

Because of the cost and the time to be performed, the spirometer assessment was randomly performed in a subsample of Look‐up 7+ project participants.

Spirometer analysis was performed according to recommendations from the American Thoracic Society guidelines.^22^ Measurements were made using the AirSmart system (https://www.nuvoair.com/). Participants were seated comfortably in a chair using a nose clip. Each subject completed tests three times with satisfactory curves; the largest FVC, FEV1, and PEF values were collected. Participants were asked to inhale to the point of maximal inspiration and exhale as quickly as possibly over the following 6 s. Effort during measurement was evaluated using a flow‐volume curve.

### Statistical analyses

Continuous variables were expressed as mean ± standard deviation (SD), and categorical variables as frequencies by absolute value and percentage (%) of the total. Descriptive statistics were used to describe demographic and key clinical characteristics of the study population according to gender. The differences in proportions and the means of covariates between the genders were assessed using Fisher's exact test and *t*‐test statistics, respectively.

The correlation between the pulmonary parameters and physical performance tests was calculated using Pearson's correlation, and the limits of agreement between measures were assessed by Blant–Altman plots. Graph insets indicate Pearson's *r* correlation values and *P* bilateral significance values (*r* and *P*, respectively). Additionally, to explore the power of FVC, FEV1, and PEF for predicting sarcopenia status, receiver operator characteristic (ROC) curves were plotted, and the area under the curve (AUC) reported, along with sensitivity and specificity at the threshold that maximized the sum of sensitivity and specificity (Youden's index).

All analyses were performed using spss software (version 11.0, spss Inc., Chicago, IL).

## Results

The mean age of 925 subjects participating in the Longevity check‐7+ surveys and receiving the spirometer evaluation was 55.6 years (standard deviation 14, range from 18 to 98 years), and 501 (54%) were women. Characteristics of the study population according to the physical activity are summarized in *Table* 1. As compared with male participants, female had a higher prevalence of normal body mass index and normal blood pressure (*P* < 0.001). On the contrary, female participants showed a higher prevalence of smoking habits (22% vs. 14%, respectively; *P* < 0.01). Male participants were more involved in physical activity compared with female subjects (*P* = 0.03). As expected, male showed a significant higher handgrip strength and better respiratory performance than female subjects. No difference was observed for the chair stand test.

**Table 1:** Characteristics of study population according to gender

Characteristics	Total sample (*n* = 925)	Male (*n* = 424)	Female (*n* = 501)	*P*
General and clinical characteristics				
Age (years)	55.6 ± 14.0	56.5 ± 13.9	55.3 ± 14.1	0.46
No smoking	754 (82)	362 (86)	392 (78)	<0.01
Healthy diet	594 (66)	263 (63)	331 (68)	0.09
Physically active	529 (57)	258 (61)	271 (54)	0.03
Normal BMI (<25 kg/m^2^)	483 (52)	184 (43)	299 (59)	<0.001
No hypertension	497 (51)	186 (44)	281 (56)	<0.001
No dyslipidaemia	293 (33)	121 (29)	172 (35)	0.08
No diabetes	858 (93)	389 (93)	469 (94)	0.51
Physical function measurements				
Chair stand test (s)	7.8 ± 2.2	7.7 ± 2.1	7.8 ± 2.2	0.36
Handgrip (kg)	32.1 ± 11.4	41.8 ± 8.8	24.2 ± 5.8	<0.001
Lung function measurements				
FVC (L)	4.0 ± 1.2	4.8 ± 1.0	3.3 ± 0.8	<0.001
FEV1 (L)	3.0 ± 0.9	3.6 ± 0.8	2.5 ± 0.6	<0.001
FEV1/FVC (%)	75.3 ± 7.5	75.2 ± 7.5	75.4 ± 7.5	0.77
PEF (L/s)	6.5 ± 2.5	8.1 ± 2.5	5.2 ± 1.6	<0.001

BMI, body mass index; FEV1, forced expiratory volume in 1 s; FVC, forced vital capacity; PEF, peak expiratory flow.

Data are given as number (per cent) for smoking, healthy diet, physical activity, normal BMI, hypertension, dyslipidaemia, diabetes; for all the other variables, means ± SD are reported. Healthy diet: consumption of at least three portions of fruit and/or vegetables per day. Physically active: physical exercise at least twice a week. No dyslipidaemia: serum cholesterol level of <200 mg/dL.

**Figure 1:**
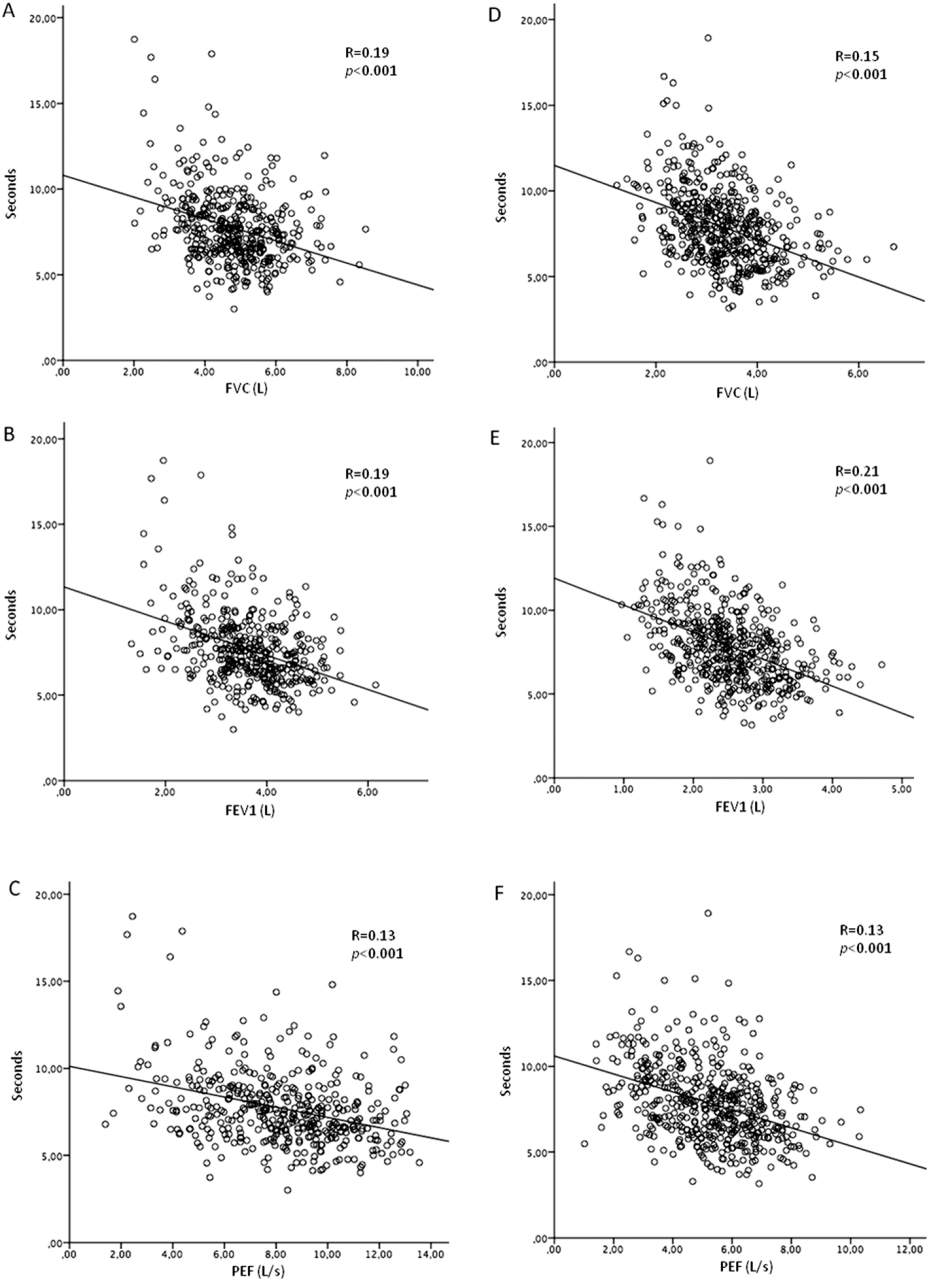
The correlation between pulmonary function and chair stand tests in male participants (Panels A, B, and C) and female participants (Panels D, E, and F). FEV1, forced expiratory volume in 1 s; FVC, forced vital capacity; PEF, peak expiratory flow.

**Figure 2:**
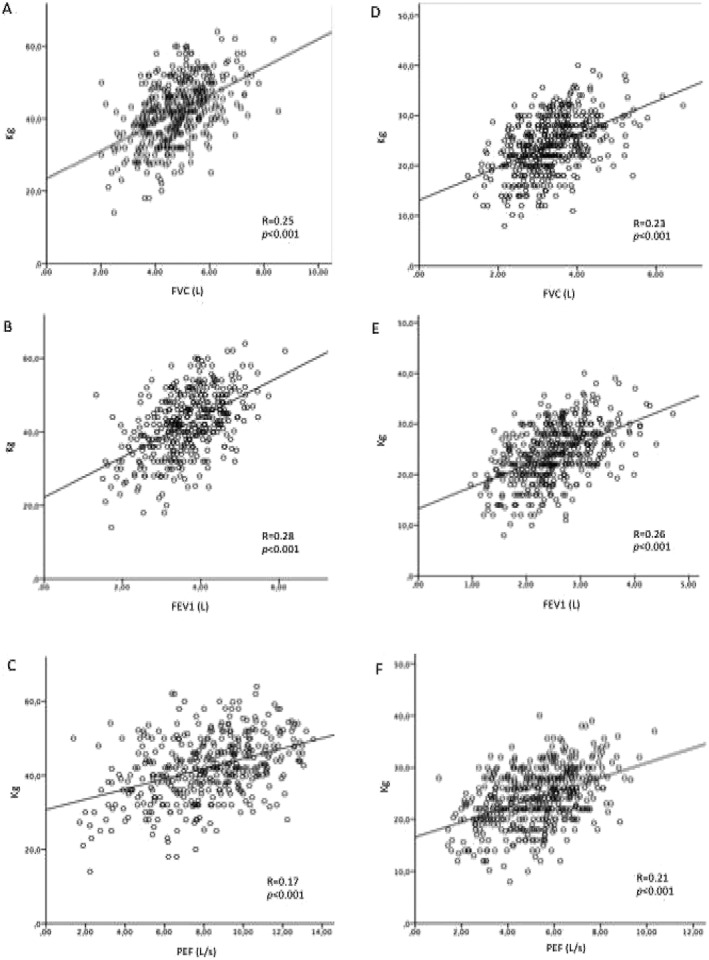
The correlation between pulmonary function and handgrip test in male participants (Panels A, B, and C) and female participants (Panels D, E, and F). FEV1, forced expiratory volume in 1 s; FVC, forced vital capacity; PEF, peak expiratory flow.

Pearson's correlation coefficients were calculated to determine the correlation between spirometer parameters and physical performance tests. *Figure* 1 shows the correlation between pulmonary function and chair stand test in male participants (Panels A, B, and C) and female participants (Panels D, E, and F). Overall, both in male and female participants, FVC, FEV1, and PEF positively correlated with chair stand test. Better results in pulmonary parameters correspond better performance in chair stand test. Similarly, FVC, FEV1, and PEF positively correlated with handgrip strength test (*Figure* 2, Panels A, B, and C for male participants and Panels D, E, and F for female participants).

**Figure 3:**
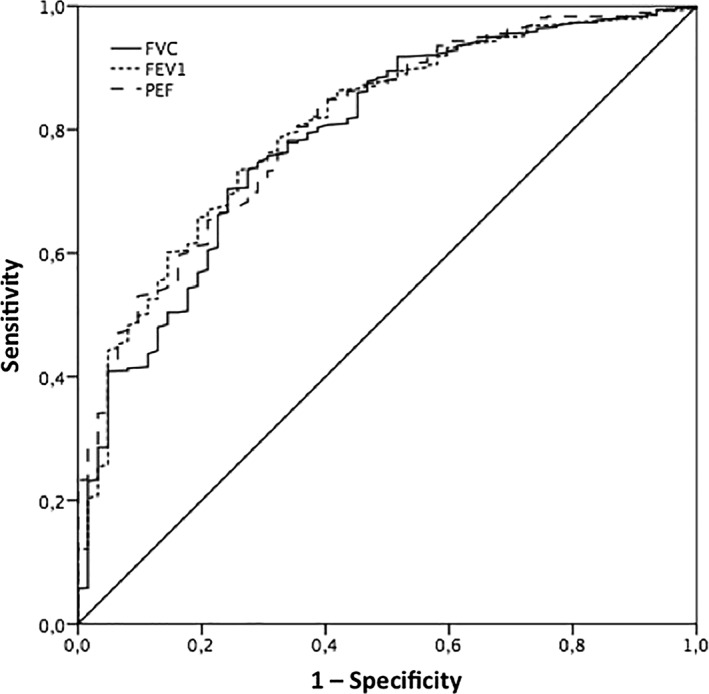
Receiver operating characteristic curve analysis for predicting sarcopenia (by means of low handgrip strength) according to pulmonary function. The receiver operating characteristic curve analysis revealed that the areas under the curves for FVC, FEV1, and PEF were 0.788, 0.804, and 0.807, respectively. FEV1, forced expiratory volume in 1 s; FVC, forced vital capacity; PEF, peak expiratory flow.

The ROC curve analysis revealed that the AUCs for FVC, FEV1, and PEF were 0.79, 0.80, and 0.80, respectively (*Figure* 3). The optimal cut‐off value in male of FVC was 3.60 L, and the sensitivity and specificity were 88% and 60%, respectively. For FEV1, the optimal cut‐off value was 2.60 L, and the sensitivity and specificity were 89% and 45%, respectively. For PEF, the optimum cut‐off value was 4.90 L, and the sensitivity and specificity were 91% and 55%, respectively. The optimal cut‐off value in female of FVC was 2.56 L, and the sensitivity and specificity were 86% and 48%, respectively. For FEV1, the optimal cut‐off value was 1.80 L, and the sensitivity and specificity were 88% and 50%, respectively. For PEF, the optimum cut‐off value was 3.40 L, and the sensitivity and specificity were 86% and 51%, respectively.

## Discussion

In the present study, we explored the correlation between respiratory parameters, physical performance, and sarcopenia in a large and unselected sample of community‐dwelling subjects without known lung disease. Our findings show that pulmonary function, assessed by FEV1, FVC, and PEF, is linearly associated with low physical performance, assessed by chair stand and handgrip strength tests. Furthermore, using the ROC analysis, the present results clearly indicate that respiratory function significantly correlated to the presence of probable sarcopenia, evaluated according to the recent EWGSOP2 consensus.^23^

A number of studies have assessed the relationships of respiratory function with muscle mass and muscle function in patients with chronic obstructive pulmonary disease (COPD).^24^, ^25^, ^26^ These previous studies were conducted in older patients in hospitals and/or nursing homes, not in community‐dwelling subjects, and had mostly small sample sizes. Nevertheless, few studies have explored lung function in healthy subjects.^25^, ^27^, ^28^ Our survey is the first assessing pulmonary and physical function in a general and in a large sample of non‐selected population. In this respect, the Look‐up 7+ project provided a unique opportunity to assess this relationship among subjects of any age and gender entered exhibition hall and/or shopping centre to visit stands and not to do a lifestyle interview and a health evaluation.

This is the first largest study demonstrating that FVC, FEV1, and PEF are positively associated with handgrip strength and chair stand tests, and the positive association found between them can be applied to apparently healthy community‐dwelling people. Specific mechanisms could elucidate the significant relationships between pulmonary function and physical performance. Respiratory muscle strength has a significant role in the respiratory system, which regulates the interaction between lung function and the respiratory muscles to maintain sufficient ventilation.^29^ Independent of specific pulmonary diseases, subjects with reduced lean body mass may have weakened capacity to inflate and deflate their lungs. Hence, the values of FVC, FEV1, and PEF can decline.

In this study, FVC, FEV1, and PEF appeared as important parameters associated with the presence of probable sarcopenia. Considering the recent EWGSOP2 consensus that defines probable sarcopenia as low muscle strength and its severity on physical performance, pulmonary function could be considered in the sarcopenia screening and assessment algorithm. For example, PEF is the maximum speed of expiration, which is usually used to monitor patients with obstructive lung diseases such as COPD and asthma. However, in healthy subjects without definite pulmonary diseases, PEF correlates with the lung volume as well as the expiratory muscle strength and power.^28^ In fact, some studies showed that PEF values increased by specific expiratory muscle strength training.^29^ Hence, our finding of FVC, FEV1, and PEF related to physical performance and sarcopenia is substantially consistent with previous studies. These three pulmonary parameters assessed by the AirSmart spirometer system (https://www.nuvoair.com/) could represent a rapid and convenient method for screening lung function in healthy subjects and in subjects with probable sarcopenia.^30^ Hence, measuring FVC, FEV1, and PEF could be useful for the screening of lung dysfunction in asymptomatic adults with decreased skeletal muscle performance.

Albeit dealing with a highly relevant issue, our study presents several limitations that need to be discussed. First, the results shown in this paper were obtained from a cross‐sectional survey limiting the ability to draw cause‐and‐effect implications between different levels of FVC, FEV1, and PEF with physical performance and sarcopenia. As a consequence, the ability to recognize whether pulmonary function is correlated with physical function and the onset of sarcopenia over time is limited. A deeper understanding of the correlation between pulmonary function and sarcopenia requires the analysis of prospective data that are not available at this stage for our study. Second, the type of evaluation could influence the assessment of some health metrics, pulmonary function, and physical performance. For example, even though the pulmonary assessment and physical performance were measured according to standard protocols, people who decided to participate in the study procedures were involved—before being assessed—in usual activities, such as walking, carrying bags, and eating. The activities, performed immediately before being evaluated, could have influenced the assessment. Limitations also include lack of information about conditions, such as asthma, COPD, and other pulmonary disease that have a direct impact on FVC, FEV1, and PEF. However, for the type of participants recruited in the study, it is possible to exclude that acute illnesses were present at the time of evaluation. Finally, the population included only Caucasian persons, so our results may not be applicable to other ethnic groups.

Apart from these limitations, this study offers a unique opportunity to investigate the pulmonary function and physical performance among an unselected population. The results clearly show that pulmonary function was positively associated with handgrip strength and chair stand tests. Given the health implications of pulmonary function, timely detection of lower handgrip strength and impaired chair stand test may be useful in assessment of potential pulmonary function impairment.

In conclusion, based on this observation, muscle strength, physical performance, and pulmonary function should be recommended as the method of choice for the early detection of individuals at risk of probable sarcopenia and at the same time to better characterize the severity of sarcopenia status.^27^, ^31^, ^32^ Finally, despite extensive research efforts, the mechanism responsible for the interaction between muscle strength, muscle function, and pulmonary performance has not been completely elucidated. A greater understanding of the mechanisms that impair muscle quality may reveal targets for intervention aimed at improving mobility and preventing disability in late life.

## Funding

The Look‐up 7+ project was supported by Italia Longeva, Marche Region, Ferrarini, Tedaldi, Fileni, Elanco, Danone Italia, MSD Italia, Carni Sostenibili, and Novartis. The study was also partly supported by intramural research grants from the Univerità Cattolica del Sacro Cuore (D3.2 2013 and D3.2 2015) to F. L. and by the non‐profit research foundation ‘Centro Studi Achille e Linda Lorenzon’.

## Author contributions

All of the authors participated in the conceiving, design, and writing of the manuscript. All authors read and approved the final manuscript. The authors of this manuscript certify that they comply with the ethical guidelines for authorship and publishing in the *Journal of Cachexia, Sarcopenia and Muscle*.^33^

## Conflict of interest

The authors declare that they have no conflict of interest.
